# Fuzzy and Sample Entropies as Predictors of Patient Survival Using Short Ventricular Fibrillation Recordings during out of Hospital Cardiac Arrest

**DOI:** 10.3390/e20080591

**Published:** 2018-08-09

**Authors:** Beatriz Chicote, Unai Irusta, Elisabete Aramendi, Raúl Alcaraz, José Joaquín Rieta, Iraia Isasi, Daniel Alonso, María del Mar Baqueriza, Karlos Ibarguren

**Affiliations:** 1 Department of Communications Engineering, University of the Basque Country (UPV/EHU), 48013 Bilbao, Spain; 2 Research Group in Electronic, Biomedical and Telecommunication Engineering, University of Castilla-La Mancha (UCLM), 16071 Cuenca, Spain; 3 BioMIT.org, Electronic Engineering Department, Universitat Politécnica de Valencia (UPV), 46022 Valencia, Spain; 4 Emergency Medical System (Emergentziak-Osakidetza), Basque Health Service, 20014 Donostia, Spain

**Keywords:** ventricular fibrillation, defibrillation, shock outcome prediction, out-of-hospital cardiac arrest, entropy measures, fuzzy entropy, sample entropy, cardiopulmonary resuscitation

## Abstract

Optimal defibrillation timing guided by ventricular fibrillation (VF) waveform analysis would contribute to improved survival of out-of-hospital cardiac arrest (OHCA) patients by minimizing myocardial damage caused by futile defibrillation shocks and minimizing interruptions to cardiopulmonary resuscitation. Recently, fuzzy entropy (FuzzyEn) tailored to jointly measure VF amplitude and regularity has been shown to be an efficient defibrillation success predictor. In this study, 734 shocks from 296 OHCA patients (50 survivors) were analyzed, and the embedding dimension (*m*) and matching tolerance (*r*) for FuzzyEn and sample entropy (SampEn) were adjusted to predict defibrillation success and patient survival. Entropies were significantly larger in successful shocks and in survivors, and when compared to the available methods, FuzzyEn presented the best prediction results, marginally outperforming SampEn. The sensitivity and specificity of FuzzyEn were 83.3% and 76.7% when predicting defibrillation success, and 83.7% and 73.5% for patient survival. Sensitivities and specificities were two points above those of the best available methods, and the prediction accuracy was kept even for VF intervals as short as 2s. These results suggest that FuzzyEn and SampEn may be promising tools for optimizing the defibrillation time and predicting patient survival in OHCA patients presenting VF.

## 1. Introduction

Out-of-hospital cardiac arrest (OHCA) is a major health problem. Yearly estimates of OHCA cases treated by Emergency Medical Systems (EMS) are over 250,000 [1] in Europe and over 180,000 in the United States [2]. Cardiac arrest is characterized by the sudden and unexpected interruption of the mechanical activity of the heart and of spontaneous breathing. This cessation of oxygen transport to the vital organs, especially to the brain, causes death within a few minutes. The most common arrhythmia causing OHCA is ventricular fibrillation (VF) [3], a non-perfusing rhythm characterized by rapid and chaotic electrical impulses, causing uncoordinated contraction of the ventricles, the main pumping chambers of the heart. The only effective way to revert VF and restore spontaneous circulation (ROSC) is to deliver an electric shock using a defibrillator [4].

During VF, the myocardium rapidly deteriorates, and early therapy is, therefore, critical. In the absence of electrical therapy, cardiopulmonary resuscitation (CPR), consisting of ventilations and chest compressions, is key to partially maintaining oxygenated blood flow to the vital organs [5,6]. In fact, CPR is key to prolonging the window of opportunity for survival until defibrillation is available. Survival rates decrease by as much as 7–10% for every minute that defibrillation is delayed if CPR is not provided, and by 3–4% when CPR is provided [7]. Once the defibrillator is available, the device analyzes the patient’s electrocardiogram (ECG), and if the arrhythmia recognition system detects VF, a shock is delivered [8,9]. Unfortunately, many defibrillator attempts are futile, resulting in deterioration of the rhythm or recurring VF [10]. Unsuccessful shocks may cause damage to the myocardium and produce unnecessary interruptions to CPR during analysis of the rhythm, charging of the defibrillator and delivery of the shock. All these factors negatively affect survival [11,12], hence the need to develop non-invasive methods to predict defibrillation success. Such methods are generally based on VF waveform analysis [13], and in most studies, shock success is defined as the presence of a rhythm with visible QRS complexes within a minute after the shock [14,15], as seen in a normal ECG. However, a temporary improvement in rhythm does not always result in survival of the patient. In fact, only around 40% of patients that recover from ROSC are discharged alive from hospital [1]. Consequently, in this study, we analyze shock success using both the classical electrocardiographic criterion and clinical criterion, i.e., the survival of the patient to hospital discharge in good neurological state.

Over the years, many ECG-based VF waveform analysis features have been introduced as shock outcome predictors [13,14,16,17]. These features are generally computed using an artifact-free ECG segment prior to the shock and exploit some of the typical characteristics of VF in its early stages, such as larger amplitudes and higher fibrillation frequencies [18] or a more complex waveform [19]. Classical features include the time domain characterization of amplitude [20,21,22], power [23] or slope [24], and spectral domain features [24,25,26]. The most extensively studied predictor, the amplitude spectrum area (AMSA), is a weighted sum of the amplitudes in the spectral domain [25,27]. The analysis of VF waveform complexity to predict shock success has been approached using classical methods based on non-linear dynamics, including the fractal dimension [19], Hurst exponent [19,28], scaling exponents [29,30], detrended fluctuation analysis [31] or Poincaré plot analysis [32]. Multi-domain approaches using machine learning techniques to combine several predictors and increase accuracy have also been explored [24,33,34,35]; however, accuracy increases have been marginal [17,24,33]. In essence, most of the defining characteristics associated with the early stages of VF are captured by features that provide compound quantification of the amplitude, spectral distribution, and complexity of the VF waveform [17]. Recent studies have shown that survival can also be predicted using AMSA or the median slope [25,36,37].

Quantitative measures of the VF waveform entropy have been recently proposed as shock outcome predictors, including the wavelet entropy [33], spectral entropy [17], and approximate entropy (ApEn) [38]. In an earlier contribution [39], we showed that regularity-based entropy measures are most useful for shock outcome prediction. In particular, ApEn and its derivatives, sample entropy (SampEn) [40] and fuzzy entropy (FuzzyEn) [41], showed better shock outcome prediction accuracy than complexity-based measures like permutation or conditional entropy. Ref. [39] used amplitude dependent matching pattern thresholds, since VF-amplitude carries information on the metabolic state of the heart [20]. This modification allowed the use of SampEn and FuzzyEn as shock outcome predictors and is in line with changes introduced in other applications in which amplitude was one of the key features explaining the time-series’ dynamics [42,43].

This is the first study to evaluate the prediction of OHCA survival using VF waveform analysis based on SampEn and FuzzyEn. Furthermore, our results show that SampEn and FuzzyEn outperform the rest of the predictors proposed to date, and that entropies should be parametrized differently depending on the criterion used for shock success. In addition, we show that SampEn and FuzzyEn are reliable shock outcome predictors with time series as short as 2 s, even when predicting the survival of the patient. Finally, we conduct an analysis on the evolution of entropy in long VF intervals without CPR therapy (20–30 s) using the pre-shock periods before defibrillation, and show that the deterioration in the characteristics of VF is not as large as predicted in previous studies [44,45].

The paper is organized as follows. Section 2 introduces the dataset that was compiled and annotated for the present study, and describes the entropy measures and the statistical methods used. The main results on shock outcome prediction for different success criteria, sequence lengths, and its evolution during pre-shock pause are reported in Section 3, in which a comparative assessment with the currently available methods is also reported. Finally, the importance of the main findings of this study is contextualized in Section 4.

## 2. Materials and Methods

### 2.1. Data Collection and Labeling

Data were collected from OHCA cases treated by the Basic Life Support (BLS) ambulances of the Basque Autonomous Community (Spain) in the period between October 2013 and December 2017. The Basque emergency medical system (EMS) is organized as a two-tier EMS system with a dense network of non-medicalized BLS ambulances, supported by fewer advanced life support ambulances (ALS) located closer to the most densely populated areas. BLS ambulance personnel provide CPR therapy and defibrillation using automated external defibrillators (AED). The system serves a population of 2.2 million, with a yearly incidence of EMS-treated OHCA of 39.1 cases per 100,000 inhabitants [46]. Electronic files recorded from the AEDs in the BLS ambulance personnel were saved in a common repository, and were later associated with the data from the cardiac arrest registry of the Basque Autonomous Region. This registry data is routinely compiled in the standard cardiac arrest reporting format (Utstein style) [47], and includes information from the emergency medical system’s coordination centers, ambulances, and hospitals, and follow-up information about the patients discharged alive from hospital.

**Figure 1:**
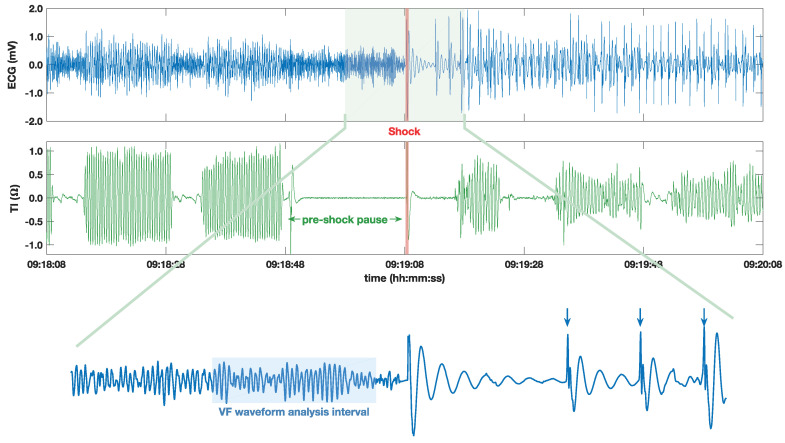
Example of the 2-min time period around a shock labeled as successful using the electrocardiographic criterion. An electrocardiogram (ECG) (**top**) was used to assess the rhythm and annotate the post-shock rhythm. The impedance (**middle**) shows chest compression activity and was used to determine the pre-shock pause interval. The ECG around the shock (**bottom**) shows how the shock restored a rhythm with sustained QRS complexes, indicated by arrows.

In the study period, over 3600 EMS treated OHCA cases were entered into the cardiac arrest registry. In 67 of those, a BLS ambulance was the first resource on scene, and in 1560 cases, AED files were available and revised. Cases in which defibrillation shocks were delivered and in which uninterrupted ECG and thoracic impedance signals were available were included in the final dataset which included 296 cases. As shown in Figure 1, thoracic impedance is necessary to monitor CPR-related chest compression activity. All patients were treated with Lifepak 1000 defibrillators (Physio-Control, Redmond, WA, USA) which have an ECG bandwidth of 0.5–21 Hz, a resolution of 4.8 μV per least significant bit, and a sampling frequency of 125 Hz. Defibrillator data, including signals and device messages, were imported to MATLAB using Physio-Control’s LIFENET Research tool and a custom made conversion tool, and all signals were resampled to 250 Hz. Shocks were identified using the device messages and were thereafter revised by two experienced biomedical engineers to certify that the pre-shock rhythm was VF and to annotate shock success according to widely accepted classical electrocardiographic criterion. Successful shocks were defined as those presenting a post shock rhythm with sustained QRS complexes at a rate of at least 30 beats per minute within a minute of the shock [15,25]. Figure 1 shows an example of a successful shock. Finally, patient outcomes were determined using the registry data, and success was defined clinically if the patient was discharged alive from hospital with a good to fair Cerebral Performance Category (CPC) score of 1–3 [48]. There were 734 annotated shocks in the final dataset. In 180 shocks (117 patients), rhythms with QRS complexes were restored and in 554 (225 patients), they were not. In total, 92 shocks were delivered to the 50 patients who survived, and 642 shocks were administered to the 246 patients who did not survive. These subgroups of shocks were further used to evaluatethe VF waveform shock outcome predictors according to (a) the electrocardiographic criterion of shock success (QRS complexes); and (b) the clinical criterion of shock success (CPC 1–3 at discharge).

### 2.2. Shock Outcome Predictors

Shock outcome predictors were computed using the VF waveform prior to the shock, leaving a 1 s guard interval for the calculation, as shown in Figure 1. Before the VF waveform analysis, the ECG was filtered to fit the typical AED bandwidth (0.5–30 Hz) using an order 8 elliptic filter with equiripple stop-band and pass-band attenuations of 30 dB and 1 dB, respectively. As in our previous contribution [39], a 5 s signal interval prior to the shock was used to compute the shock outcome predictors, although in the additional analyses based on SampEn and FuzzyEn, the effect of using different interval lengths was explored.

#### 2.2.1. Shock Outcome Predictors from the Literature

For this study, twenty five VF waveform shock outcome predictors were implemented, covering the published methods in the field. Details on the calculations can be found on the original references, and the MATLAB code with our implementation is available in the following online resource: https://github.com/BChicote/shockOutcome. The time domain features included the amplitude range (AR) [21], peak-to-peak amplitude (PPA) [14], mean amplitude (MA) [18], signal integral (SignInt) [21], two definitions of the VF waveform root mean square (RMS) value, RMS1 [17] and RMS2 [31], mean and median slope (MS and MdS) [24], and a smoothed nonlinear energy operator (SNEO) [49]. The spectral domain features were based on a 2048 point fast fourier transform (FFT) of the hamming windowed analysis interval, and comprised the AMSA [50], centroid frequency (CF), dominant or peak frequency (PF), energy (ENRG), spectral flatness measure (SFM) [26], centroid power (CP), maximum power (MP), and power spectrum analysis (PSA) [24]. The VF waveform complexity measures derived from non-linear dynamics were the Hurst exponent (Hu) [28], the scaling exponent (ScE) [16], the logarithm of the absolute correlations (LAC) [30], two coefficients derived from detrended fluctuation analysis (DFA1 and DFA2) [31] and the median stepping increment (MSI) derived from the Poincaré plot analysis [32], in addition to two entropy measures: wavelet-entropy (WE) [33] and spectral entropy (SEN) [17].

#### 2.2.2. Quantification of VF Waveform Regularity-Based on Entropy Measures

In a previous contribution, we showed that the measures of entropy chosen to quantify VF waveform regularity are good shock outcome predictors—in particular, SampEn and FuzzyEn [39]. These measures quantify the regularity of a time series by evaluating repetitive patterns along the ECG segment. They are extensions of the approximate entropy (ApEn), introduced by Pincus [51], and overcome some of its shortcomings, such as the dependence of ApEn on the length of the analysis interval or its relative lack of consistency. SampEn, defined by Richman and Moorman, differs from ApEn in that it does not count self matches and does not use a template wise approach [40]. FuzzyEn is an extension of SampEn introduced by Ref. [41] in which vector matches are defined in a smooth way using the fuzzy set theory [41]. To compute SampEn and FuzzyEn, signals were resampled to $f_{s}^{*}=60$ Hz (see Appendix B for generalized time-delayed entropies and the influence of the sampling frequency), which is compatible with the AED bandwidth used in the study and lowers the computational cost.

To compute SampEn, the samples of the signal in the analysis interval, x(n), were decomposed into i=1,…,N−m+1 vectors of size *m*, where *N* is the number of samples in the interval. This yielded vectors of the form $x_{i}^{m}=\left\{x\left(i\right),x\left(i+1\right),\ldots ,x\left(i+m-1\right)\right\}$. The distance between two vectors, $x_{i}^{m}$ and $x_{j}^{m}$, was measured using the maximum norm ($L_{\infty }$-norm):

(1) $$d_{ij}^{m}=\max_{k=0,\ldots ,m-1}\left(|x(i+k)-x(j+k)|\right).$$

Vector matches were counted using the Heaviside function, $\Theta \left(x\right)=\frac{1}{2}+\frac{1}{2}\mathrm{sign}\left(x\right)$, as the membership function, and a tolerance (*r*) for the matches in the following way:

(2) $$C_{i}^{m}\left(r\right)=\frac{1}{N-m-1}\sum_{j=1,j\neq i}^{N-m}\Theta \left(r-d_{ij}^{m}\right),$$

where j≠i prevents self matches. The probability that two vectors of length *m* match with tolerance *r* is then:

(3) $$\phi^{m}\left(r\right)=\frac{1}{N-m}\sum_{i=1}^{N-m}\ln C_{i}^{m}\left(r\right).$$

The process iwa repeated for vectors of length m+1:

(4) $$\begin{array}{c} C_{i}^{m+1}\left(r\right)=\frac{1}{N-m-1}\sum_{j=1,j\neq i}^{N-m}\Theta \left(r-d_{ij}^{m+1}\right), \end{array}$$

(5) $$\begin{array}{c} \phi^{m+1}\left(r\right)=\frac{1}{N-m}\sum_{i=1}^{N-m}C_{i}^{m+1}\left(r\right). \end{array}$$

Then, SampEn was estimated as

(6) $$\mathrm{SampEn}\left(m,r,N\right)=\ln\phi^{m}\left(r\right)-\ln\phi^{m+1}\left(r\right).$$

FuzzyEn is similar to SampEn, but before evaluating vector match counts, the baseline is subtracted from the $x_{i}^{m}$ vectors, so match counts are based on the local characteristics of the signal. The vectors used to compute the $L_{\infty }$-norm distances using Equation (1) were

(7) $${\bar{x}}_{i}^{m}=\left\{x\left(i\right),x\left(i+1\right)\ldots ,x\left(i+m-1\right)\right\}-\frac{1}{m}\sum_{l=0}^{m-1}x\left(i+l\right),$$

and instead of using a binary membership function, Θ(x), matches were determined using a family of exponentially decaying functions, $D_{ij}^{m}\left(n,r\right)=\exp\left(-{\left(d_{ij}^{m}/r\right)}^{n}\right)$. In this paper we used n=2 and a Gaussian distance of $D_{ij}^{m}\left(2,r\right)=\exp\left(-{\left(d_{ij}^{m}/r\right)}^{2}\right)$, as proposed in [52]. So, the equations for the match counts were

(8) $$\begin{array}{c} C_{i}^{m}\left(r\right)=\frac{1}{N-m-1}\sum_{j=1,j\neq i}^{N-m}D_{ij}^{m}\left(2,r\right),\;C_{i}^{m+1}\left(r\right)=\frac{1}{N-m-1}\sum_{j=1,j\neq i}^{N-m}D_{ij}^{m+1}\left(2,r\right). \end{array}$$

The probabilities of vectors of lengths *m* and m+1 matching for tolerance, *r*, $\phi^{m}\left(r\right)$ and $\phi^{m+1}\left(r\right)$, were calculated using Equations (3) and (5), and FuzzyEn was estimated as

(9) $$\mathrm{FuzzyEn}\left(m,2,r,N\right)=\ln\phi^{m}\left(r\right)-\ln\phi^{m+1}\left(r\right).$$

### 2.3. Optimal Parameters for SampEn and FuzzyEn

In the context of shock outcome prediction, the VF amplitude contains relevant information on the metabolic condition of the myocardium [20]. Consequently, the matching tolerance (*r*) is not normalized to the standard deviation of the signal [39]. In most studies, typical values of m=1,2 and r∈ (0.05–0.25) times the standard deviation of the signal are used, although these values may be inappropriate in some applications [53]. That is why in the first stage we explored the optimal (m,r) combinations for SampEn and FuzzyEn using a 30×20 search grid with m=1,2,3 and r=5,10,...,100 μV. Furthermore, we used two distinct and relevant definitions of shock success: a temporary reversal of the arrhythmia, evidenced by sustained QRS complexes, and a clinical criterion based on survival in good neurological state. Consequently, the optimal (m,r) pairs for SampEn and FuzzyEn may differ depending on the criterion used for shock success.

### 2.4. Evaluation of Shock Outcome Prediction

Shock outcome prediction is a binary decision problem, in which the positive class is shock success. Therefore, a 2×2 confusion matrix can be built to compare the clinical annotations and the decisions based on the predictors, and to obtain the sensitivity (Se) for successful shocks and the specificity (Sp) for unsuccessful shocks. Furthermore, by varying the decision threshold for the predictor, a receiver operating characteristics (ROC) curve can be constructed. Then, the area under the curve (AUC) can be used to evaluate the predictive power of the VF-waveform features [54]. In this study, the optimal point in the ROC curve was defined as the one that maximized the balanced accuracy (BAC):

(10) $$\mathrm{BAC}=\frac{1}{2}\left(\mathrm{Se}+\mathrm{Sp}\right),$$

that is, Se and Sp are weighted equally. This is equivalent to the Youden index, although alternative ROC cut-off points have been defined [55]. The identification of potentially beneficial shocks and avoidance of unnecessary shocks that may cause myocardial damage and prolonged CPR interruptions are both important.

### 2.5. Complementary Analyses

According to the resuscitation guidelines [56], CPR should be interrupted every 2-min to evaluate the rhythm in an artifact free segment and to determine if a shock should be delivered. The minimization of interruptions in CPR is important to improve survival [12], so shock outcome prediction methods should use a signal interval that is as short as possible to make a decision. Furthermore, if VF analysis intervals as short as 2–3 s could be used to predict shock success, these methods could be deployed during the ventilation pauses in 30:2 CPR, where 30 chest compressions are followed by 2 rescue breaths that normally last 4–6 s. Several studies have shown that these ventilation pauses can be accurately identified using the AED impedance channel and that VF can be identified during those pauses [57,58]. Since dominant frequencies in human VF are in the 3–7 Hz range, short analysis intervals of 2 s should be sufficient to capture the dynamics of VF.

In addition, it is well known that long interruptions in CPR therapy are detrimental for the survival of the patient [12,45]. In particular, pre-shock pauses should be shortened as much as possible. However, it is not known whether the dynamics of VF, as captured by shock outcome predictors, deteriorate during pre-shock pauses to reflect the impact of interrupting CPR therapy in survival. Consequently, in this study we also analyzed the evolution of shock outcome predictors along the pre-shock pause, and quantified the deterioration in entropy as therapy progresses through a regression analysis.

## 3. Results

### 3.1. Optimal Parameters to Compute Entropy Measures

**Figure 2:**
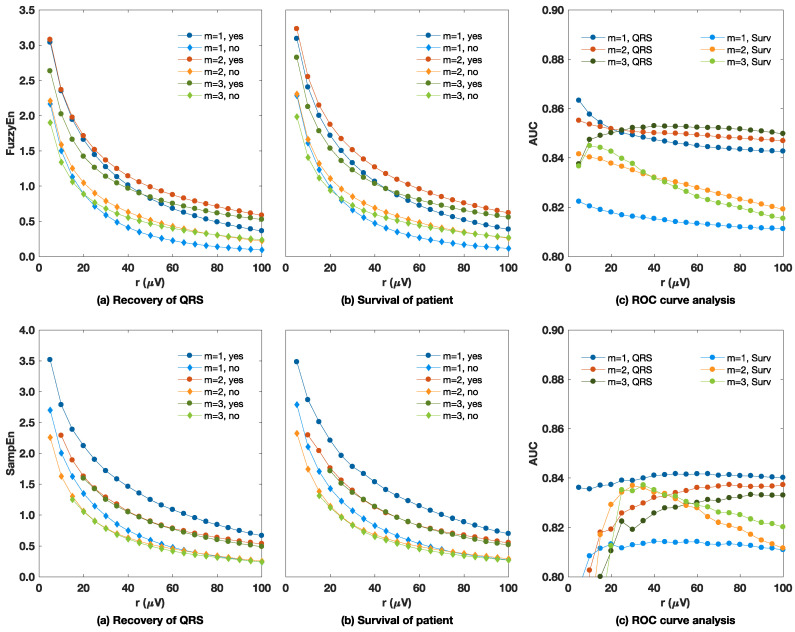
Analysis of the optimal (m,r) values for fuzzy entropy (FuzzyEn) (**top**) and sample entropy (SampEn) (**bottom**) using a 5 s analysis interval. Both success criteria were analyzed separately, and the optimal ranges to predict shock success were derived from the receiver operating characteristics (ROC) curve analyses (right) and are summarized in Table 1.

An accurate estimation of a time series regularity using SampEn and FuzzyEn involves the proper selection of *m* and *r* [59]. Figure 2 summarizes the experiments to determine the optimal (m,r) values or ranges of values for shock outcome prediction, and the optimal ranges derived thereof are reported in Table 1. The figure shows how the median values of FuzzyEn and SampEn changed as the matching tolerance (*r*) increased. As expected, the entropies decreased as the matching tolerance increased for all values of *m*. Furthermore, entropies for unsuccessful shocks (for both success criteria) were smaller than for successful shocks, which shows that more regular VF is less amenable to defibrillation. This finding is in line with previous studies [38,39], although it had never been assessed to predict the survival of the patient. Furthermore, for all the (m,r) combinations studied, the median values of entropy for successful shocks were significantly higher than for unsuccessful shocks, with p<0.0001 for the Mann–Whitney test. The ROC curve analysis shown in the right panels of Figure 2 shows that a temporary improvement in prognosis (QRS criterion) is easier to predict than the long term survival of the patient which may be affected by multiple factors besides electrical therapy. In addition, the optimal ranges for the calculation of the entropies are different if a temporary improvement or the long term survival are to be predicted, as shown in the values reported in Table 1. Our findings on this data are consistent with our previous contribution [39] in which, for a different and more limited dataset, the optimal (m,r) values for FuzzyEn and SampEn using the electrocardiographic criterion were found to be (3, 80 μV) and (1, 50 μV), respectively. The optimal values reported in Table 1 are used in the paper for the rest of the analyses. Furthermore, with these definitions, entropy measures were used to capture the non-linear nature of VF, as shown by the surrogate data testing reported in Appendix A.

### 3.2. Comparison with Other Shock Outcome Predictors

**Figure 3:**
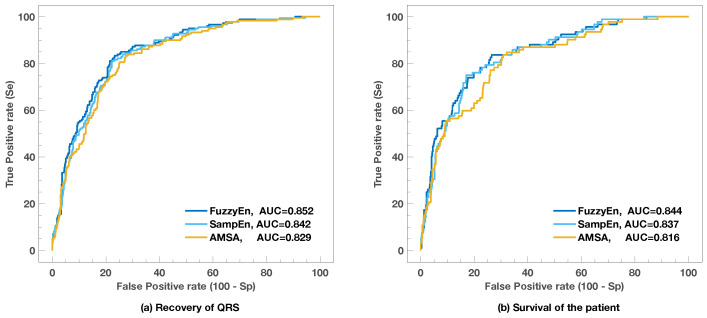
ROC curves for AMSA compared to entropy measures for both success criteria.

Figure 3 and Table 2 compare the ROC curves and their critical cut-off points for FuzzyEn, SampEn, and AMSA. The latter is the best accepted shock outcome predictor and is customarily used as the reference predictor [15,17,31,38,39]. The ROC curve analysis shown in Figure 3 reveals that, in our data, entropy measures were more accurate than AMSA for all possible combinations of Se and Sp for both success criteria. The Se and Sp values obtained for four critical points in the curve are shown in Table 2. This includes working points to avoid unnecessary shocks; Se for high Sp to avoid missing beneficial shocks; Sp for high Se; and the optimal point in the ROC curve according to Youlden’s index or to the point closest to the (0,1) point in the curve [55].

When compared to the rest of the predictors available in the literature, the ROC curve analysis again revealed that FuzzyEn and SampEn are the best predictors, as shown in Table 3. This held for both success criteria.

### 3.3. Shortening the Analysis Interval

**Figure 4:**
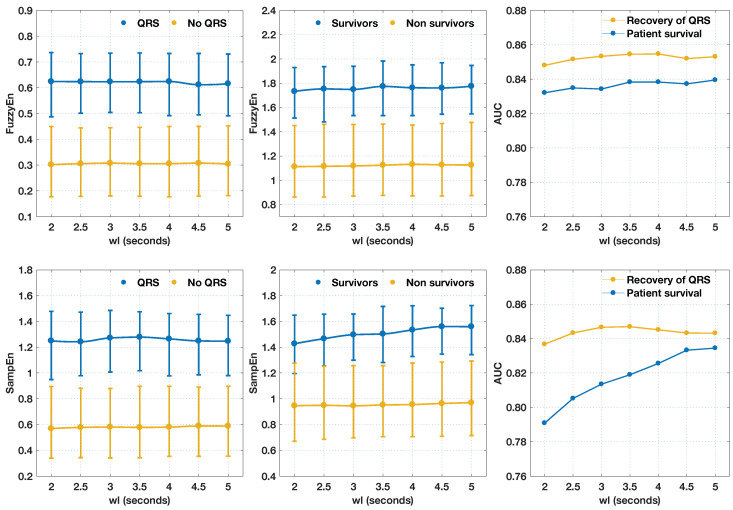
Values of FuzzyEn (top) and SampEn (bottom) computed using different window lengths (wl). The length of the interval in samples was $N_{w}=f_{s}\cdot \mathrm{wl}=60\cdot$ wl for each case. The AUC values for each segment length and the two outcome criteria are shown in the rightmost graphs. FuzzyEn and SampEn were computed using the optimal (m,r) pairs obtained for 5-s segments, as reported in Table 1.

Shortening the analysis interval may be critical in some shock outcome prediction scenarios, for instance, if the analysis of the VF waveform is performed in the ventilation pauses during CPR [58]. Such situations may require analysis intervals as short as 2–3 s. Figure 4 shows that the values of FuzzyEn and SampEn for the two outcome groups as the analysis interval (see Figure 1) shortened from 5 to 2 s. The values of entropy remained stable as the analysis interval shortened. The differences in the values of entropy when QRS or survival was used as outcome criteria were due to the different (m,r) pairs used in each case. Interestingly, FuzzyEn was more robust as the analysis interval shortened, and its predictive power remained stable for both criteria with very small variations in AUC (under 1-point) for all analysis intervals. On the contrary, the predictive power of SampEn for survival considerably degraded for analysis intervals below 3.5 s. In fact, AUC values ranged from 0.837 for 5 s analysis intervals to 0.791 for 2 s analysis intervals, a drop of almost five points in AUC. This only happened when survival was used as the outcome variable; the variation in the recovery of QRS complexes was below 1.2 points for all analysis intervals.

**Figure 5:**
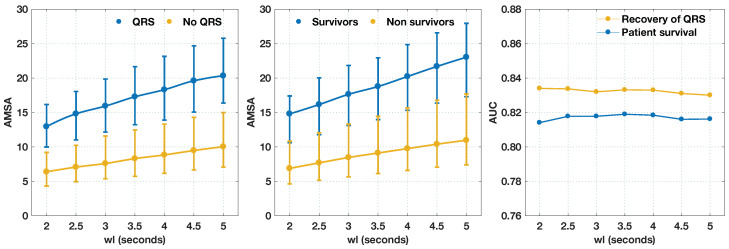
Values of AMSA and its predictive power for different window lengths (wl), and the two outcome criteria.

Dependence on the window length was also analyzed for AMSA, and the results are shown in Figure 5. As shown in the figure, AMSA retained its predictive power for small window lengths, a result that confirms recent findings [37]. When compared to FuzzyEn, the figure shows that AMSA is a worse predictor than FuzzyEn for all window lengths and both outcome criteria, and it only outperformed SampEn for survival with short window lengths.

### 3.4. Evolution of Entropy Values During the Preshock Pause

**Figure 6:**
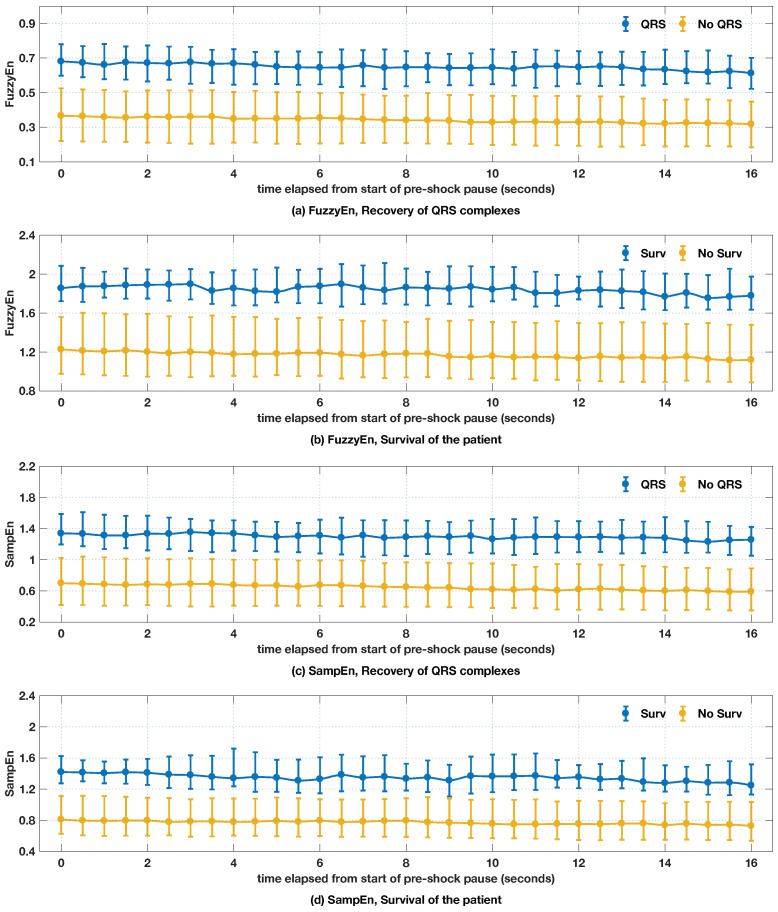
Evolution of entropies during the pre-shock pause, disaggregated for the different outcomes. t=0 s corresponds to the start of the pre-shock pause. In all cases, entropies decreased (almost) linearly as the interval without chest compression therapy increased. Entropies were computed using 3 s intervals, and values were computed every 0.5 s during the pause.

Longer pre-shock pauses have been associated with lower probability of shock success [45] and lower survival rates [12]. It is therefore possible that the VF waveform shows deterioration during the pre-shock pause that can reflect the absence of oxygen supply (through CPR therapy) and this could be measurable in terms of shock outcome predictors; some older evidence points in this direction [44]. Our data contained 734 shocks with a median (10–90 percentile) pre-shock pause duration of 21.8 (17.9–27.7) s. We found significant differences at the 95% level in pre-shock pause duration between survivors 20.5 (17.7–28.4) s (*n* = 92 pauses, 50 patients) and non-survivors 21.9 (18.0–27.6) s (*n* = 646 pauses, 249 patients) , p=0.03 (Mann–Whitney test). The evolution of the entropies during the pre-shock pause (see Figure 6) showed a linear decrease in entropy as the period without CPR therapy was prolonged; however, this decline was very small. Furthermore, there was a clear separation in the entropy values between successful and unsuccessful shocks, regardless of the entropy measure or outcome criterion selected. This separation was maintained throughout the pre-shock pause for durations as long as 16s which shows that if there is a deterioration in VF dynamics during the pre-shock pause, this is not shown by the entropy values measured along the pause.

The linear regression analysis for the trends in Figure 6 is shown in Table 4. Furthermore, differences in slopes and intercepts, i.e., the regression coefficients of the linear regression analyses for successful and unsuccesful shocks were compared using an extension of the t-test described in Weaver and Wuensch [62]. In all cases, there was a significant difference in the intercept but no significant difference in the slope. That is, the predictors separated both classes (intercept), but the deterioration in VF dynamics measured by entropy was similar for successful and unsuccessful shocks (slope). In any case, this deterioration was very small. For example, using FuzzyEn and the recovery of QRS as a success criterion, pre-shock pauses of 1 min 25s would be needed to go from the average FuzzyEn of 0.664 (succesful) to 0.389 (unsuccessful). These are much longer than the normal pre-shock pauses seen in OHCA, which are in the range of 10–30 s [12,45]. In our data, the entropy shock outcome predictors showed that the VF waveform dynamics do not considerably deteriorate during the pre-shock pause, and that this deterioration is similar for survivors and non-survivors.

## 4. Discussion and Conclusions

Having optimal timing for defibrillation delivery would contribute to an improved survival rate of OHCA patients by avoiding or minimizing interruptions in chest compression therapy and potential myocardial damage caused by futile and repetitive shocks [15,25]. Defibrillation success is associated with VF waveform characteristics, such as amplitude, dominant frequency, and waveform regularity [20,63]. We hypothesized that properly chosen entropy measures could be used for the combined characterization of VF amplitude and waveform regularity, therefore allowing accurate prediction of defibrillation success and patient survival. Our results, based on a cohort of almost 300 OHCA patients, support this hypothesis. Furthermore, we showed that these regularity-based entropies can be effectively used with minimal interruptions to CPR therapy by shortening the analysis intervals to 2 s, and evaluated VF waveform degradation during long intervals of VF without CPR therapy. The present study showed that FuzzyEn and SampEn are the best predictors of defibrillation success and patient outcome when compared to the available methods in the literature. Furthermore FuzzyEn outperformed all available methods, even for very short analysis intervals, so entropy based shock outcome prediction could become the method of choice in the future. In addition, the code with our implementation of the methods is available online (https://github.com/BChicote/shockOutcome), for researchers to test our results and conduct future experiments using other datasets.

VF with larger amplitudes are more responsive to defibrillation, and are associated with the so called electrical (<4 min) and circulatory (4–10 min) phases of VF [64]. The amplitude conveys relevant information about the phase of VF and the state of the myocardium, and entropy measures have to be modified to account for amplitude [42,43]. Consequently, our computation of SampEn and FuzzyEn used amplitude-dependent matching tolerances and produced a combined characterization of VF amplitude and regularity, as introduced in an earlier contribution [39]. The most accurate predictors of defibrillation success directly measure, or are dependent on, VF amplitude [14], including methods based on indices derived from non-linear dynamics [17,39]. Larger entropy values are associated with defibrillation success, a result that confirms earlier findings using ApEn [38], SampEn, and FuzzyEn [39]. However, although successful shocks result in a temporary improvement in the condition of the patient, evidenced by a rhythm with better prognosis, very often the rhythm rapidly reverts to VF (recurrent VF) or even asystole [10]. Furthermore, only about 40% of patients that recover from spontaneous circulation during treatment survive [1]. For instance, in our data, from the 115 patients that presented ROSC at any time during resuscitation, 113 were admitted alive to hospital, but only 50 survived. It is therefore important to evaluate the extent to which entropy measures predict survival. Our results confirm that survival can be predicted with accuracies similar to those of defibrillation success with survivors presenting larger values of entropy in the pre-shock VF intervals (see Figure 2). Some recent evidence is in line with this finding, since AMSA has been shown to predict survival (hospital discharge) and long term survival of patients (6 month to 1 year follow-up) [25,36,37]. In our case, follow-up data was not available, so we could only assess survival to hospital discharge, although these two outcomes are strongly correlated with survival at 6 month follow-up above 85% [25]. Interestingly, the AUCs for survival using AMSA in two of the studies [36,37] were lower than in our case (0.72 and 0.75, compared to 0.81). Our results, however, are similar to the ones reported in Ref. [25]. Survival rates in our data were similar to those in Ref. [25] (17% and 19%), and significantly lower than those in the other two studies which were, in both cases, above 35%. So, there seems to be an association between higher survival rates and lower predictivity that needs to be further investigated and that may be probably associated with lower predictability of survival in the earlier phases of VF when survival is more likely.

The consumption of oxygen and energy in the myocardium is higher during VF because the ventricles activate and contract at a higher frequencies [65]. Increased energy consumption occurs in the non-ischemic heart in the early phases of VF [66], and it is prolonged even during CPR, resulting in decreased creatine-phosphate (CrP) levels and contractibility [67]. These depleted CrP levels compromise the recycling of adenosine triphosphate (ATP) in the myocites, and may impair the contractility of the sarcomeres which require high energy or ATP levels. ATP levels decrease significantly during prolonged VF [68], decreasing the likelihood of restoration of a perfusing rhythm which is associated with higher ATP levels [69]. During the electrical phase of VF, ATP levels decrease moderately, and in the circulatory phase, partial reperfusion through CPR may temporarily increase ATP levels close to the pre-arrest levels [70]. Concurrent measurement of ATP levels and quantitative measures of the VF waveform have shown direct relationships between ATP levels and AMSA, MS, or ScE [71], with positive relationships for measures with a positive relationship to defibrillation amenability (AMSA and MS) and negative relationships in the opposite case (ScE). There is also a linear association between increases in AMSA or MS and an increase in coronary perfusion pressure [72], a surrogate measure of cardiac perfusion. These relationships have not been directly determined for FuzzEn; however, there is a strong association between most efficient shock outcome predictors including AMSA-FuzzEn (positive) and AMSA-ScE (negative), as shown in Appendix C.

The characterization of SampEn and FuzzyEn in terms of *m* and *r* shows that the prediction of defibrillation success and patient survival is very stable (see AUCs in the rightmost panels of Figure 2), so the selection of (m,r) is not critical for prediction accuracy. Defibrillation success can be predicted more accurately than survival, although the differences in AUC for wide ranges of (m,r) values were below two points. It is noteworthy that patient survival was better predicted with smaller values of *r*. Our interpretation is that survival is not as dependent on large differences in VF amplitude as amenability to defibrillation. By using smaller values of *r*, small amplitude changes are more relevant, so FuzzyEn can more accurately quantify differences in pattern matches for lower amplitude VF. Waveform irregularity may play a more important role in patient survival, although the physiological mechanism that explains this result is not clear. This observation was confirmed by the drop in AUC (see Table 3) in the parameters that directly quantify VF amplitude, such as peak-to-peak amplitude (PPA), mean amplitude (MA), and signal integral (SigInt). In all these cases, the AUC dropped by 5–6 points for the prediction of survival, while the AUC drops only by 1–2 points for features that incorporate additional information, such as AMSA (the spectral content of high frequency bands) and MSI (based on Poincaré plot analysis). Finally, it is noteworthy that FuzzyEn marginally outperformed SampEn by 0.7–1 points in AUC. Using a soft boundary to determine pattern matches resulted in a more precise estimation of entropy, since entropy can change continously rather than at fixed steps (SampEn) [52]. In addition, FuzzyEn was not ill defined for small values of *r* which sometimes produces no matches in hard boundary cases (SampEn), an effect that was more noticeable as the segment length decreased.

Predicting defibrillation success with very short signal intervals may be critical, particularly if these methods are used during ongoing CPR. Although some studies based on AMSA and related predictors show that intervals as short as 0.8–1 s are sufficient for the prediction of shock success [37,38], in practice, those intervals will never be shorter than 2–3 s. An interval of at least 3 s is needed to accurately identify that the patient’s rhythm is VF [9,58,73], and this has to be done before VF waveform analysis based on AMSA or FuzzyEn is applied. Furthermore, CPR is customarily delivered in sequences of 30 chest compressions followed by 2 ventilations, namely 30:2 CPR. During chest compressions, the VF waveform is corrupted by movement artifacts [74], but it can be accurately detected and reliably analyzed during ventilations [57,58]. Furthermore, if the defibrillator is charged during chest compressions, the rhythm analysis interval to detect VF can be shortened to around 3 s [73]. All these scenarios require short VF waveform analysis intervals, preferably under 3 s, and our results confirm that entropies used to characterize VF waveform can be reliably estimated with signal segments as short as 2 s. There was a drop in performance when SampEn was used to predict survival for shortened signal segments (see Figure 4). In our analyses, we used SampEn with m=2 and r=25μV, but as the analysis interval shortened, such low values of *r* produced, in some cases, very few matches and an ill-valued SampEn. This explains the drop of almost five points in AUC (bottom right figure). Again, FuzzyEn was shown to be robust even in these challenging scenarios, and should be used instead of SampEn for the prediction of defibrillation success and patient survival. This comes at an increase in computational cost associated with the evaluation of the fuzzy membership function and the inapplicability of some recently developed efficient implementations of SampEn [75]. Although current AED hardware is low-end, it is sufficient for the computational cost of FuzzyEn for sequences of 120 (2s, $f_{s}^{*}=60$ Hz) to 300 (5 s) samples.

Finally, our data allowed us to evaluate the evolution of VF waveform in long periods without CPR, using entropies as surrogate measures. This was possible because the pre-shock pauses in our data were long (median duration >20 s), although typical of AED use [76]. We did observe significant differences in pre-shock pause duration between survivors and non-survivors, in line with some recent findings [12]. However, entropies degraded very slowly during the pre-shock pause, an interval in which the lack of chest compressions interrupts the re-oxygenation of the myocardium. Although VF increases cardiac oxygen consumption by over 70% compared to a normal rhythm [67], this is not immediately reflected in the VF waveform deterioration and a decrease in entropy. In our data, it was shown that periods as long as 90 s may be required to considerably reduce entropy values from those observed in successful shocks to those observed in unsuccessful shocks (if the linear trend observed in Figure 6 is mantained). This deterioration seems to be independent of the condition of the myocardium, since no significant differences were observed between VF corresponding to successful and unsuccessful shocks (or survivors and non-survivors). Very long intervals for VF waveform deterioration have also been observed from the onset on VF in pigs [63,77].

Cardiac arrest remains difficult to predict. There is evidence suggesting that heart rate variability, as a non-invasive measure of cardiac autonomic dysfunction, could be used to predict cardiac arrest using indexes derived from the temporal, spectral, and non-linear analyses of interbeat series [78,79]. If reliable, such heart rate variability indices could be incorporated as a life saving tools into bedside monitors [80]. However cardiac arrest prediction based on heart rate variability is sub-optimal [79,81], and considering that, in most cases, cardiac arrest occurs as a first clinical event or to subjects at very low risk, it is difficult to foresee how it could be applied to the population at large [3]. So, the best science for the prevention and treatment of cardiac arrest is compiled in the resuscitation guidelines [7], in which quantitative measures of VF waveform for shock outcome prediction are gradually gaining importance [25].

Our study had some limitations. First, data came from a single type of device, the LP1000 AED. Although the ECG acquisition bandwidth and ECG amplitude resolution may differ between devices, the data available for the study was acquired using values similar to those in most commercial devices [39]. Our results should be replicated with data from other devices, but we do not expect relevant differences. Second, our data were retrospective and the effectiveness of these methods could be better assessed in prospective studies [15]; however, data from prospective studies on shock outcome prediction is not available to date. Third, our data came from OHCA, so several factors that affect VF waveform and patient survival could not be assessed, such as the VF duration prior to AED placement or the quality of CPR delivered by bystanders. Differences in electrode placement and prior patient conditions, such as ischemia or medications could not be determined [15,82].

## Figures and Tables

**Table 1:** Optimal parameters to compute entropies depending on the criterion used for success. For the matching tolerance (*r*) both a wide range of values and the optimal points are reported.

	Criterion for Success	
	Electrical	Clinical
**SampEn**		
Range	m=1, r=20–100μV	m=2, r=20–40μV
Optimum	m=1, r=50μV	m=2, r=25μV
**FuzzyEn**		
Range	m=3, r=25–90μV	m=3, r=10–20μV
Optimum	m=3, r=80μV	m=3, r=15μV

**Table 2:** Comparison of the ROC curves in Figure 3 for four critical points of the ROC curve.

	ROC Cutoff Point			
	Se ${}^{†}$	Sp ${}^{‡}$	Se/Sp (J)	Se/Sp (0,1)
**Electrical**				
FuzzyEn	55.0	56.1	83.3/76.7	81.1/78.5
SampEn	51.1	57.9	81.1/77.6	81.1/77.6
AMSA	44.4	52.2	83.3/72.6	80.6/74.9
**Survival**				
FuzzyEn	55.4	49.7	83.7/73.5	76.1/80.2
SampEn	53.3	52.2	75.0/83.0	75.0/83.0
AMSA	54.3	45.2	83.7/77.2	77.2/74.0

${}^{†}$ for sensitivity (Se) = 90%, ${}^{‡}$ for specificity (Sp) = 90%.

**Table 3:** Shock outcome predictor performance ranked by the area under the curve (AUC), and the optimal point (Youden’s index) for which Se, Sp and Balanced Accuracy (BAC) are reported.

Recovery of QRS				
			**Optimal Point**	
**Feature**		**AUC**	**BAC**	**Se/Sp**
FuzzyEn	[39]	0.852	80.0	83.3/76.7
SampEn	[39]	0.842	79.4	81.1/77.6
MdS	[24]	0.839	78.0	81.7/74.4
MSI	[32]	0.838	78.4	82.2/74.5
MS	[24]	0.836	78.0	83.9/72.2
PPA	[60]	0.833	77.6	87.2/68.1
SNEO	[49]	0.829	77.1	77.8/76.4
AMSA	[50]	0.829	77.9	83.3/72.6
PSA	[24]	0.829	77.3	90.0/64.6
ScE	[29]	0.812	75.5	87.8/63.2
AR	[21]	0.797	76.0	87.2/64.8
ENRG	[24]	0.797	75.0	89.4/60.5
RMS1	[17]	0.794	75.3	82.8/67.9
RMS2	[31]	0.794	74.5	84.4/64.6
MA	[18]	0.793	74.6	90.6/58.7
SigInt	[22]	0.793	74.6	90.6/58.7
LAC	[30]	0.765	71.4	75.6/67.3
MP	[24]	0.764	71.0	72.8/69.3
CP	[24]	0.759	71.0	73.3/68.6
DFA2	[31]	0.731	69.8	63.3/76.4
Hu	[28]	0.729	69.1	71.1/67.1
PF	[26]	0.724	70.0	76.1/63.9
CF	[26]	0.688	66.8	81.7/52.0
WE	[61]	0.683	66.3	78.9/53.8
SFM	[26]	0.658	62.8	54.4/71.1
SEN	[17]	0.644	61.6	61.7/61.6
DFA1	[31]	0.532	55.2	46.7/63.7
**Survival of the Patient**				
			**Optimal Point**	
**Feature**		**AUC**	**BAC**	**Se/Sp**
FuzzyEn		0.844	78.6	83.7/73.5
SampEn		0.837	79.0	75.0/83.0
MSI		0.816	76.1	81.5/70.7
MdS		0.816	76.3	81.5/71.0
AMSA		0.816	76.4	83.7/69.2
MS		0.812	76.0	81.5/70.6
PPA		0.788	74.6	82.6/66.7
PSA		0.781	73.5	88.0/58.9
SNEO		0.779	72.5	85.9/59.0
ScE		0.748	70.3	84.8/55.8
AR		0.739	70.6	88.0/53.1
ENRG		0.735	70.7	85.9/55.5
RMS1		0.735	69.4	79.3/59.5
RMS2		0.734	69.4	79.3/59.5
MA		0.734	69.6	79.3/59.8
SigInt		0.734	69.6	79.3/59.8
WE		0.728	69.6	66.3/72.9
CF		0.717	68.3	73.9/62.6
LAC		0.701	65.5	78.3/52.8
PF		0.698	66.7	57.6/75.7
MP		0.695	65.6	59.8/71.3
Hu		0.691	65.4	59.8/71.0
CP		0.686	64.7	67.4/62.0
DFA2		0.652	63.7	52.2/75.2
DFA1		0.593	57.8	64.1/51.6
SFM		0.544	55.4	22.8/88.0
SEN		0.534	54.3	54.3/54.2

**Table 4:** Regression analysis of the evolution of FuzzyEn and SampEn during the pre-shock pause, from the beginning of the pause (interruption of CPR) until 16 s, using a 3 s interval for the computation of entropies with values computed every 0.5 s.

	Recovery of QRS			Survival of Patient		
	No	Yes	*p*-Value	No	Yes	*p*-Value
**FuzzyEn**						
Intercept	0.389	0.664	<0.01	1.280	1.917	<0.01
Slope (min^−1^)	−0.167	−0.194	0.54	−0.398	−0.362	0.74
**SampEn**						
Intercept	0.761	1.334	<0.01	0.866	1.417	<0.01
Slope (min^−1^)	−0.351	−0.385	0.72	−0.279	−0.432	0.15

**Figure A1:**
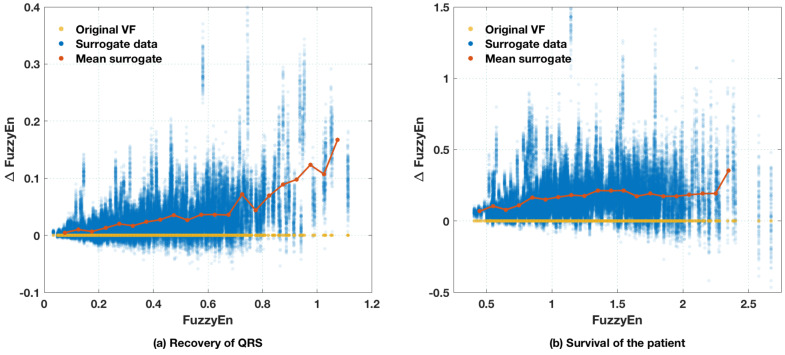

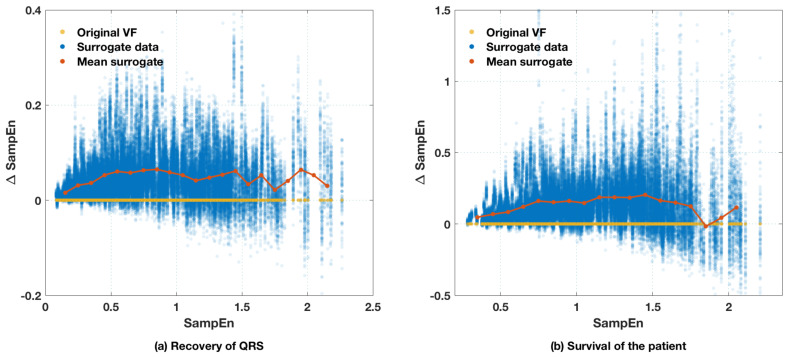
Entropy values of the original VF signals (baseline), and the difference in entropy with respect to baseline for the 100 surrogate signals created using the amplitude adjusted fourier transform (AAFT) method. The red line shows the mean trends of the difference in entropy with respect to baseline of the surrogate data. The entropy values are significantly higher for the surrogate test signals for FuzzyEn (**top**) and SampEn (**bottom**) computed for the optimal values to discriminate recovery of QRS (**left**) or survival of the patient (**right**).

**Figure A2:**
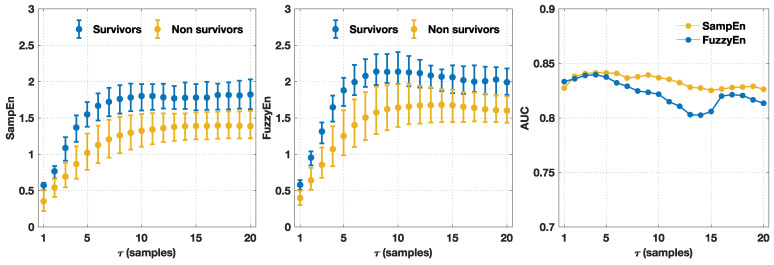
Values of SampEn(m,r,τ,N) and FuzzyEn(m,2,r,τ,N) and their predictive powers for signals sampled at $f_{s}=250$ Hz. All calculations were done for the optimal points for prediction of survival.

**Figure A3:**
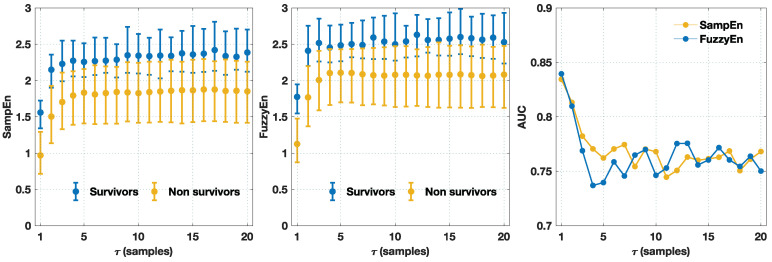
Values of SampEn(m,r,τ,N) and FuzzyEn(m,2,r,τ,N) and their predictive powers for signals sampled at $f_{s}=60$ Hz. All calculations were done for the optimal points for prediction of survival.

**Figure A4:**
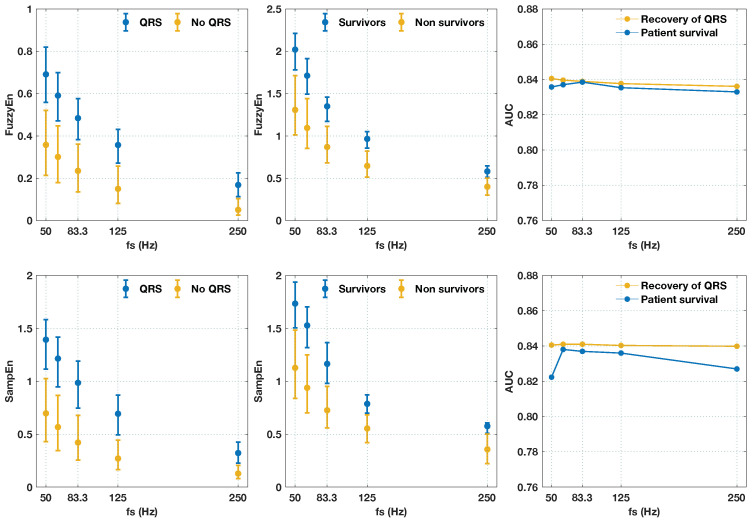
Values of the entropies and their predictive power as a function of the sampling frequency. All values were computed for the optimal (*m*, *r*) pairs reported in Table 1.

**Figure A5:**
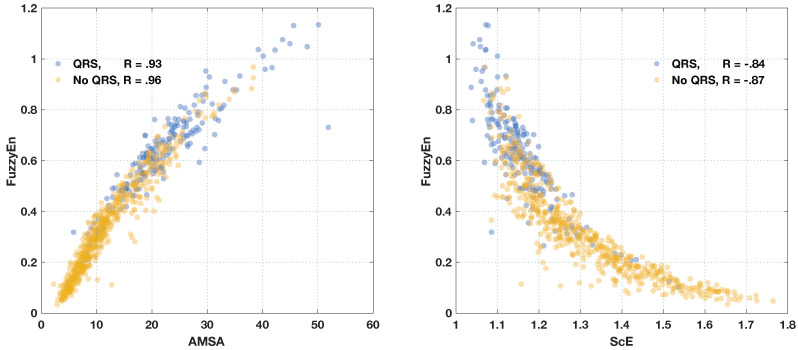
Relation between FuzzyEn and the typical VF waveform measures for which associated ATP level values and CPP values exist. As shown in the figure there is a strong positive association between AMSA and FuzzyEn for succesful and unsuccessful shocks (total R=0.96), and a strong negative association between ScE and FuzzyEn (total R=−0.87).

## Appendix A. Surrogate Data Testing

An important question is whether the entropy measures introduced in the paper indeed captured the non-linear properties of the VF waveform. Surrogate data analysis [83,84] gives a statistical framework to test such hypothesis. In our analysis, we created 100 surrogate signals for each of the original VF waveforms using the amplitude adjusted fourier transform (AAFT) method [83,84]. The surrogate signals preserved the second-order linear structure of the original signal (autocorrelation function) and its amplitude distribution. SampEn and FuzzyEn were computed for the surrogate signals using the optimal values from Table 1 and were compared to the value obtained for the original signal. A one sided t-test was done to determine whether the surrogate signals presented a significantly higher (p<0.05) value than VF under analysis. The results of the surrogate data testing are shown in Figure A1.

As shown in the figure, the surrogate signals presented higher values of entropy for SampEn and FuzzyEn, regardless of the optimal (*m*, *r*) point selected for their calculation (maximize prediction of recovery of QRS or survival of the patient). When analyzed individually, the mean value of entropy for the surrogate dataset was significantly larger (p<0.05) than that of the original signal in more than 93.7% (SampEn) and 90.5% (FuzzyEn) of cases when the recovery of QRS definitions were used, and in 92.5% (SampEn) and 98.7% (FuzzyEn) of cases for the survival of the patient. With these results we conjecture that the non-linear nature of VF is better captured when lower thresholds *r* are used (see Table 1).

## Appendix B. Time Delayed Entropies and Sampling Frequency

The analysis based on SampEn and FuzzyEn can be generalized to time series with different time delays as proposed by Kaffashi et al. [85]. In this manner long range correlations in the signals can be explored. Instead of taking consecutive samples from the signals, we could introduce a time delay (τ) to form vectors of the form

(A1) $$\begin{array}{c} x_{i,\tau }^{m}=\left\{x\left(i\right),x\left(i+\tau \right),\ldots ,x\left(i+\left(m-1\right)\tau \right)\right\}, \end{array}$$

(A2) $$\begin{array}{c} {\bar{x}}_{i,\tau }^{m}=\left\{x\left(i\right),x\left(i+\tau \right)\ldots ,x\left(i+\left(m-1\right)\tau \right)\right\}-\frac{1}{m}\sum_{l=0}^{m-1}x\left(i+l\tau \right). \end{array}$$

for i=1,2,⋯N−mτ. The distance between vectors is then

(A3) $$d_{ij}^{m}=\max_{k=0,\ldots ,m-1}\left(|x(i+k\tau )-x(j+k\tau )|\right)\;\mathrm{or}\;d_{ij}^{m}=\max_{k=0,\ldots ,m-1}\left(|\bar{x}\left(i+k\tau \right)-\bar{x}\left(j+k\tau \right)|\right).$$

Vector matches are counted using a membership function (M(·)) and a tolerance (*r*) for the matches in the following way

(A4) $$C_{i}^{m}\left(r\right)=\frac{1}{N-m\tau -1}\sum_{j=1,j\neq i}^{N-m\tau }M\left(r,d_{ij}^{m}\right)\;\mathrm{and}\;C_{i}^{m+1}\left(r\right)=\frac{1}{N-m\tau -1}\sum_{j=1,j\neq i}^{N-m\tau }M\left(r,d_{ij}^{m+1}\right),$$

where the membership function is either $\Theta (r-d_{ij}^{\ell })$ for SampEn, or $D_{ij}^{\ell }\left(2,r\right)=\exp\left(-{\left(d_{ij}^{\ell }/r\right)}^{2}\right)$ for FuzzyEn (in our analysis n=2), and *ℓ* is *m* or m+1. The probability that two vectors of length *m* or two vectors of length m+1 match with tolerance *r* is then

(A5) $$\phi^{m}\left(r\right)=\frac{1}{N-m\tau }\sum_{i=1}^{N-m\tau }\ln C_{i}^{m}\left(r\right)\;\mathrm{and}\;\phi^{m+1}\left(r\right)=\frac{1}{N-m\tau }\sum_{i=1}^{N-m\tau }C_{i}^{m+1}\left(r\right),$$

and the time delayed entropies are computed similarly by applying $\ln\phi^{m}\left(r\right)-\ln\phi^{m+1}\left(r\right)$ (Equation (6) or (9)) to yield SampEn(m,r,N,τ) or FuzzyEn(m,2,r,N,τ), depending on the choice of vectors and membership functions.

In order to study the possible long range correlations, entropies were computed for the original sampling frequency of $f_{s}=250$ Hz and delays of τ=1,2⋯,20. The results for the optimal (m,r) pairs to discriminate patient survival (see Table 1) are shown in Figure A2. Entropy values increased as τ increased until they plateaued for τ∼7, but the discriminative power decreased for values of τ above 6. For the same tolerance (*r*) for smaller time delays (closer samples), more matches are likely for vectors of size *m* and m+1, so entropies were smaller. The results for the time-delayed entropies for $f_{s}=60$ Hz, the sampling frequency used in the manuscript, are shown in Figure A3. In this case, increasing τ had a large negative impact on the predictability of our entropy measures, and the entropies plateaued for smaller values of τ. Intuitively, lowering the sampling frequency is related to using larger delays, since this consecutive samples to be separated by larger sampling periods.

A related question is the influence of the sampling frequency on the predictability of the entropy measures. To keep the analysis simple, we considered the original signal, x(n), of *N* samples with a sampling frequency $f_{s}=250$ Hz. Given that our data was filtered using the typical AED bandwidth of 0.5–30 Hz, the signal was oversampled and could therefore be decimated without information loss to sampling frequencies down to 60 Hz (in practice given the spectral content of VF recorded by surface defibrillation pads, a sampling frequency of 50 Hz is tolerable). In such cases, the decimated signal was related to the original signal by an integer time delay factor τ:

(A6) $$x_{d}\left(n\right)=x\left(\tau n\right),\;n=1,2,\cdots ,\frac{N}{\tau }\;\mathrm{and}\;\tau \in N.$$

Then, the equations from the main manuscript could be applied to the decimated signals to obtain the entropy estimates. This analysis is related to the time-delayed entropies but with a fundamental difference. In the time-delayed entropies [85], matches are found for the vectors $x_{i,\tau }^{m}$ with i=1,2,⋯,N−mτ, but in those vectors, consecutive samples from the original signal are separated by τ samples. When the original definitions are applied to the decimated signals, $x_{d}\left(n\right)$, the samples in vectors $x_{i}^{m}$ are again separated by τ, but the matches are only computed for i=1,τ,2τ⋯. In both cases, we measured correlations between samples separated by the same time interval of $\tau \cdot T_{s}$, where $T_{s}$ is the sampling period, but in a fundamentally different way.

In our case, given the sampling frequencies and the bandwidth of the VF waveform, we were only able to consider τ = 1–5, which corresponds to $f_{s}=250,125,83.3,62.5,50$ Hz. The results for the two outcome groups and both entropies as a function of τ are shown in Figure A4. All data points were computed for the optimal (*m*, *r*) pairs reported in Table 1. The figure shows there was no difference in predictability when using different time delays, although the entropy estimations changed due to the varying pattern matches (as observed in Figure A2 for τ≤5). The predictive power was maintained for different $f_{s}$, and in our application, the use of smaller sampling frequencies implied a reduced computational cost which could be critical in AEDs with limited computational resources. So, a sampling frequency adjusted to the typical AED bandwidth through resampling, $f_{s}^{*}=60$ Hz as described in the main text, is a sound choice.

## Appendix C. Relations Between FuzzyEn and Other Predictors

This section provides indirect evidence of the relation between FuzzyEn and myocardial ATP levels and coronary perfusion pressure (CPP) during VF. Animal data with concurrent ATP levels or CPP measurements at different time points in VF and the corresponding FuzzyEn values is not available. However, the studies by Salcido et al [71] and Reynolds et al [72] have shown positive relationships between AMSA and ATP levels [71] and between changes in CPP and AMSA [72] and the corresponding negative relationship with ScE. Figure A5 shows the relationship of AMSA and ScE to FuzzyEn and the different Pearson correlation coefficients (*R*) for the two subgroups of data show successful and unsuccesful shocks. Given the strong positive/negative correlations between AMSA–FuzzyEn and ScE–FuzzyEn, we can safely conjecture that the results obtained by Salcido et al and Reynolds et al for the associations between ATP levels and AMSA and CPP changes and changes in AMSA would be also observed for FuzzyEn.
